# A delusion of pregnancy in man with hyperprolactinemia

**DOI:** 10.1192/j.eurpsy.2021.2031

**Published:** 2021-08-13

**Authors:** A. Aissa, H. Ghabi, D. Khattech, S. Meddouri, U. Ouali, F. Nacef

**Affiliations:** 1 Psychiatry A, Razi Hospital, Manouba, Tunisia; 2 Psychiatry A Department, Razi Hospital, Manouba, Tunisia

**Keywords:** Delusion of pregnancy, - hyperprolactinemia-, male

## Abstract

**Introduction:**

A delusion of pregnancy in men has been rarely reported in psychiatric disorders. The literature on this delusion in male schizophrenia is limited. It was reported especially in medical conditions. In psychiatric disorders, it has been explained for a long time by psychodynamic theories.

**Objectives:**

To present a case of a pregnancy delusion in man associated temporally to neuroleptic-induced hyperprolactinemia and a review of literature of medical and psychological etiologies of this symptom

**Methods:**

We presented a case of a pregnancy delusion in man associated temporally to neuroleptic-induced hyperprolactinemia and we elucidated through a review of literature of medical and psychological etiologies of this symptom.

**Results:**

Case report A 46-year-old man, unmarried, who had a mild intellectual disability and a 22-year history of schizophrenia. He was admitted to our hospital for psychotic relapse due to the interruption of his medication. This patient had been treated for years with long action injection medication. On admission he was disorganized, verbalizing a poorly-systematized fuzzy delirium. And he believed he was pregnant. Serum prolactin levels was 38 ng/ml (3-25ng/ml). He was put on Haldol decanoate 150mg/month, chlorpromazine 150mg/day, and diazepam 15mg/day.

**Conclusions:**

The presentation of a delusion of pregnancy in man is rather infrequent. The delusion may have many social, psychological, and biological determinants to its genesis. This case highlights the importance of medical investigations notably the assay of prolactin in the assessment of patients who present with delusions of pregnancy.

**Disclosure:**

No significant relationships.

